# Microbiological study of sternal osteomyelitis after median thoracotomy – a retrospective cohort study

**DOI:** 10.1186/s12879-023-08340-7

**Published:** 2023-05-25

**Authors:** Olimpiu Bota, Feras Taqatqeh, Florian Bönke, Klaus Matschke, Adrian Dragu, Stefan Rasche, Kevin Bienger, Maxime Mülhausen

**Affiliations:** 1 University Center for Orthopedics, Trauma and Plastic Surgery, Faculty of Medicine Carl Gustav Carus, TU Dresden, Fetscherstraße 74, 01307 Dresden, Germany; 2 Department of Cardiac Surgery, University Heart Center Dresden, TU Dresden, Dresden, Germany; 3 Surgical Intensive Care Unit, Faculty of Medicine Carl Gustav Carus, TU Dresden, Dresden, Germany

**Keywords:** Deep sternal wound infection, Microbiology, Sternal osteomyelitis, Cardiac surgery, *Staphylococcus aureus*, *Staphylococcus epidermidis*, Gram-negative bacteria, *Enterococcus* spp

## Abstract

**Introduction:**

Deep sternal wound infection is a rare but feared complication of median thoracotomies and is usually caused by microorganisms from the patient’s skin or mucous membranes, the external environment, or iatrogenic procedures. The most common involved pathogens are *Staphylococcus aureus, Staphylococcus epidermidis* and gram-negative bacteria. We aimed to evaluate the microbiological spectrum of deep sternal wound infections in our institution and to establish diagnostic and treatment algorithms.

**Methods:**

We retrospectively evaluated the patients with deep sternal wound infections at our institution between March 2018 and December 2021. The inclusion criteria were the presence of deep sternal wound infection and complete sternal osteomyelitis. Eighty-seven patients could be included in the study. All patients received a radical sternectomy, with complete microbiological and histopathological analysis.

**Results:**

In 20 patients (23%) the infection was caused by *S. epidermidis*, in 17 patients (19.54%) by *S. aureus*, in 3 patients (3.45%) by *Enterococcus* spp., in 14 patients (16.09%) by gram-negative bacteria, while in 14 patients (16.09%) no pathogen could be identified. In 19 patients (21,84%) the infection was polymicrobial. Two patients had a superimposed *Candida* spp. infection. Methicillin-resistant *S. epidermidis* was found in 25 cases (28,74%), while methicillin-resistant *S. aureus* was isolated in only three cases (3,45%). The average hospital stay for monomicrobial infections was 29.93 ± 13.69 days and for polymicrobial infections was 37.47 ± 19.18 (p = 0.03). Wound swabs and tissue biopsies were routinely harvested for microbiological examination. The increasing number of biopsies was associated with the isolation of a pathogen (4.24 ± 2.22 vs. 2.18 ± 1.6, p < 0,001). Likewise, the increasing number of wound swabs was also associated with the isolation of a pathogen (4.22 ± 3.34 vs. 2.40 ± 1.45, p = 0.011). The median duration of antibiotic treatment was 24.62 (4–90) days intravenous and 23.54 (4–70) days orally. The length of antibiotic treatment for monomicrobial infections was 22.68 ± 14.27 days intravenous and 44.75 ± 25.87 days in total and for polymicrobial infections was 31.65 ± 22.29 days intravenous (p = 0.05) and 61.29 ± 41.45 in total (p = 0.07). The antibiotic treatment duration in patients with methicillin-resistant Staphylococci as well as in patients who developed an infection relapse was not significantly longer.

**Conclusion:**

*S. epidermidis* and *S. aureus* remain the main pathogen in deep sternal wound infections. The number of wound swabs and tissue biopsies correlates with accurate pathogen isolation. With radical surgical treatment, the role of prolonged antibiotic treatment remains unclear and should be evaluated in future prospective randomized studies.

## Introduction

As opposed to the superficial surgical site infection (SSI), which comprises only the skin and subcutaneous tissues in any anatomical region, the deep sternal wound infection (DSWI) describes the postoperative infection after median thoracotomy which extends beyond the deep fascia and may involve the sternal bone and the mediastinal tissues [1]. This is a rare but feared complication with an incidence of 0,5 − 4% and current mortality rates of 10%, when the proper treatment is promptly initiated. The infection is usually caused by microbiological agents from the patient’s skin or mucous membranes, the external environment, or iatrogenic procedures [2]. The pathogens invade the sternal bone and surrounding mediastinal tissues, thereby preventing the surgical wound from healing [3]. The presence of bacteria in the sternal wound is usually aggravated by the patient’s comorbidities, which weaken the immunity and lead to the development of DSWI. The risk factors for developing this complication may be patient-related (diabetes, obesity, age, chronic obstructive pulmonary disease (COPD), congestive heart failure, nasal contamination with *Saphyloccoccus aureus*), surgery-related (use of both internal mammary arteries (BIMA), revision for bleeding, prolonged surgical time, retrosternal hematoma) or related to the hospital stay (prolonged ventilation, extended hospitalization) [1, 4].

Not only the appropriate treatment but also an early diagnosis is relevant for the prognosis [5]. Therefore, if diagnosis and initiation of treatment are delayed, mediastinitis, sepsis, septic shock, infection with multi-resistant pathogens, abscess formation and chronicization may occur [6]. Although the spectrum of microbiological agents varies in the literature, the most common are *Staphylococcus aureus*,*Staphylococcus epidermidis* and gram-negative bacteria (GNB). *S. epidermidis* belongs to the physiological skin flora, while *S. aureus* can be found mostly in the mucous membranes of the nose, throat and perineum. *S. aureus* can lead to purulent infections and wounds with abscess formations. It affects bone function and may lead to bone resorption [7, 8]. In comparison, *S. epidermidis* preferentially attaches to artificial surfaces such as venous catheters and produces biofilms like *S. aureus* [9]. The formation of biofilms often correlates with the failure of antibiotic treatment. Therefore, the removal of foreign bodies and prostheses and the surgical debridement of the wounds is essential. Furthermore, the development of methicillin-resistant strains can further complicate the treatment and worsen the prognosis (Chaudhuri, 2012).

The aim of our study was to identify the main pathogens and their resistance spectrum responsible for the development of DSWI at our institution, to identify possible risk factors leading to a certain infection type and to evaluate the impact of specific pathogens on the treatment course and outcome. Furthermore, we aimed at identifying the role of wound swabs and tissue biopsies in the microbiological diagnosis and to determine if the length of antiobiotic treatment influences the development of an infection relapse.

## Methods

We retrospectively evaluated the DSWI at our institution between March 2018 and December 2021. The inclusion criteria were the presence of a DSWI and a complete sternal osteomyelitis (SO). The exclusion criteria were DSWI without SO or with manubrial osteomyelitis. Eighty-seven patients could be included in the study. All patients received a radical sternectomy, with complete microbiological and histopathological analysis. The retrospective data gathering was performed in Excel 365 (Microsoft Corporation, WA, USA). The statistical analysis was performed using JASP version 0.16.4 (University of Amsterdam, The Netherlands). For nominal variables contingency tables were used and the Chi-square Test and Fisher’s exact Test were performed. The continuous data was tested for normal distribution using the Shapiro Wilk test. An independent t-test was performed with the assumption checks of normal distribution and equal variances (Brown-Forsythe Test). If the assumption checks of equal variances did not hold, the Welch Test was used and if the assumption checks of normal distribution did not hold the Mann-Whitney Test was performed. Statistical significance was assumed when p < 0.05.

The study was approved by the Ethics Committee of TU Dresden (*BO-EK-387,082,020).*

## Therapeutical strategy

After the diagnosis of DSWI with SO is established, timely surgical debridement is planned and the antibiotic treatment is paused. The soft tissues are debrided until healthy, bleeding tissue is reached, any foreign bodies are removed and the whole sternum is resected, either en bloc or piecemeal. Wound swabs are harvested after removing the dressing and when purulent secretions are encountered. At least three bone biopsies from the most affected regions are harvested and sent natively directly to microbiological examination. Finally, the pericardium, lung and mediastinal tissues are carefully debrided, and negative pressure wound therapy (NPWT) is initiated. If the parietal pleura and the pericardial cavity remain closed, instillation with dwell time (NPWTi-d) can be initiated on the first postoperative day. Three two five days later, the wound is evaluated in the operating room. If the wound is vital, beginning to granulate and without purulent secretions, the flap closure is performed. No sterile wound swabs are required, as the closure with a well-vascularized tissue will ensure the healing of the otherwise adequately debrided wound. When the wound is clinically not adequate for flap closure, further NPWT is continued with dressing changes every 3–5 days. The flap closure is standardly performed using the pedicled latissimus dorsi musculocutaneous flap. When the lateral decubitus required for this flap is not possible, the unilateral or bilateral pectoralis major muscle flap is used.

## Results

We could include 87 patients in the study. All patients suffered from DSWI with sternal osteomyelitis and received a radical sternectomy. The mean age of the cohort was 67.43 ± 7.45 and there were 20 females (22.99%). The mean Body Mass Index (BMI) was 29.54 kg/m^2^ ± 5.25. (Table 1) Nine (10.34%) patients died during treatment. Six patients died of sepsis and multiple organ failure. One patient developed a massive pulmonary artery embolism. One patient died of COVID-19 pneumonia and another patient of a bleeding brain metastasis, both not being directly related to the DSWI.

**Table 1:** General patient information and risk factors

Risk factor	Number	Percent
Age	67.43 ± 7.46	
BMI (kg/m^2^)	29.54 ± 5.25	
Male	67	77.01%
Female	20	22.99%
ASA		
3 4	71 16	81.61% 18.39%
Radical sternectomy		
Piecemeal En-bloc	65 22	74.71% 25.29%
Acute sternal osteomyelitis Chronic sternal osteomyelitis	82 5	94.25% 5.75%
Muscle flap coverage Latissimus Pectoralis major	83 80 3	95.40% 96.39% 3.61
NPWT		
no treatment simple NpWT NPWTi-d	3 36 48	3.45% 41.38% 55.17%
Smoking	34	39.08%
Alcohol	11	12.64%
Obesity	44	50.58%
Diabetes mellitus Insulin	53 25	60.92% 28.74%
Hyperlipidemia	44	50.58%
Peripheral arterial disease	19	21.84%
Asthma	3	3.45%
COPD	14	16.09%
Renal insufficiency	39	44.83%
Pulmonary artery embolism	2	2.29%
Deceased	9	10.35%

As all patients were multimorbid, the American Society of Anesthesiologists physical status (ASA) was 3 in 71 patients (81.61%) and 4 (18.39%) in 16 patients. The average hospital stay was 29 ± 15.25 days. The radical sternectomy was performed on average in 65.5 ± 30.7 min. The average number of days until a muscle flap coverage could be performed was 7 ± 6.3, and 2 ± 1.4 surgeries were performed on average until the muscle flap coverage was possible. A chronic SO was considered when the heart surgery occurred more than three months before the DSWI diagnosis. Eighty-two patients had an acute SO (94.25%) while five patients suffered from a chronic SO (5.75%). The wounds were radically debrided and NPWT was initiated until wound coverage. Thirty-seven patients (42.35%) received a normal NPWT, 48 (56.47%) received NPWTi-d with hypochlorite solution, and two died before receiving NPWT. Considering the flap coverage, 80 patients (91.95%) received a pedicled latissimus dorsi muscle flap, three patients (3.45%) a unilateral or bilateral pectoralis major muscle flap and four patients died before the wound could be closed (Table 2). We checked symptoms, laboratory and radiological findings to see if the patients suffered from pneumonia and pleural effusion before the sternectomy, after the sternectomy and after the muscle flap coverage. One (1.15%) patient had pneumonia before the radical sternectomy, six patients (6.9%) developed pneumonia after the first surgery and four patients (4.6%) had pneumonia after the muscle flap coverage. Thirteen patients (14.94%) had a pleural effusion before the radical sternectomy, 50 patients (57.47%) after the radical sternectomy and 54 patients (62.07%) after the muscle flap coverage (Table 3). Considering the intravenous antibiotic treatment 65 patients received penicillins (74.71%), 20 patients cephalosporins (22.99%), 18 patients carbapenems (20.69%), 27 patients vancomycin (31.03%), five patients quinolones (5.75%), nine patients clindamycin (10.34%), six patients daptomycin (6.9%) and four patients received gentamicin and linezolid respectively (4.6%). Considering the oral antibiotic treatment 25 patients received quinolones (28.74%), five patients penicillins (5.75%), 16 patients co-trimoxazole (18.39%) and ten patients received rifampicin (11.49%) (Table 4).

**Table 2:** Treatment information

	Mean/Median	25th percentile	75th percentile
Average Hospital Stay	29	21	40
Operative time of sternectomy (min)	65.5	55.25	83.75
Average number of days until muscle flap coverage	7	5	12
Average number of surgeries until muscle flap coverage	2	1	3
Average number of revision surgeries	1	0	1

**Table 3:** Incidence of pneumonia and pleural effusion during treatment

Pulmonary complication	Before sternectomy	After sternectomy	After flap coverage
Pneumonia	1 (1.15%)	6 (6.9%)	4 (4.6%)
Pleural effusion	13 (14.94%)	50 (57.47%)	54 (6207%)

**Table 4:** Administered intravenous and oral antibiotics

Intravenous antibiotics	Number of patients	Percent of patients
Penicillins	65	74.71
Cephalosporins	20	22.99
Carbapenems	18	20.69
Vancomycin	27	31.03
Clindamycin	9	10.34
Damptomycin	6	6.9
Quinolones	5	5.75
Gentamycin	4	4.6
Linezolid	4	4.6
**Oral antibiotics**	**Number of patients**	**Percent of patients**
Quinolones	25	28.74
Co-trimoxazole	16	18.39
Rifampicin	10	11.49
Penicillins	5	5.75

**Fig. 1:**
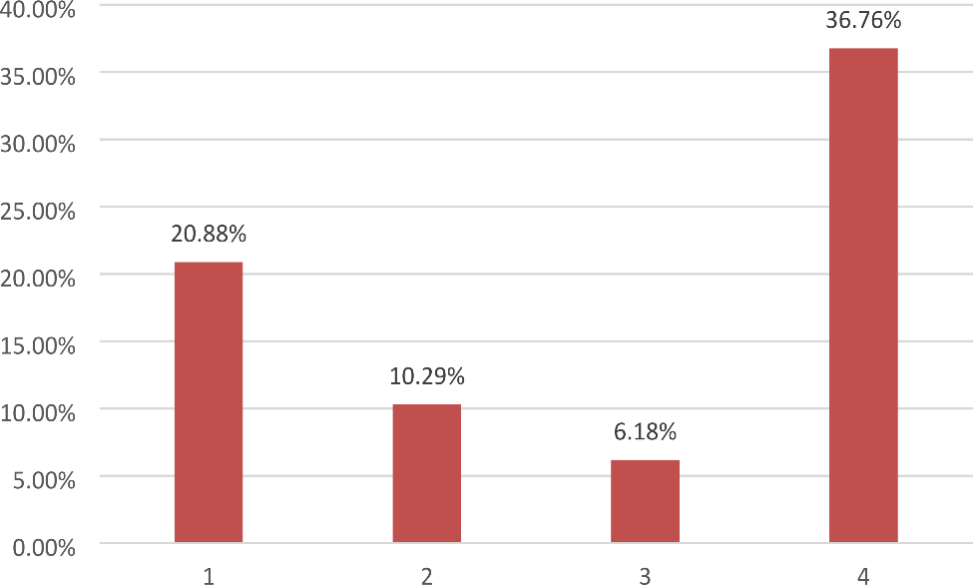
Overview of wound swabs: 1 – *Staphylococcus aureus*, 2 – *Staphyloccocus epidermidis*, 3 – Enterococci, 4 – gram-negative bacteria

**Fig. 2:**
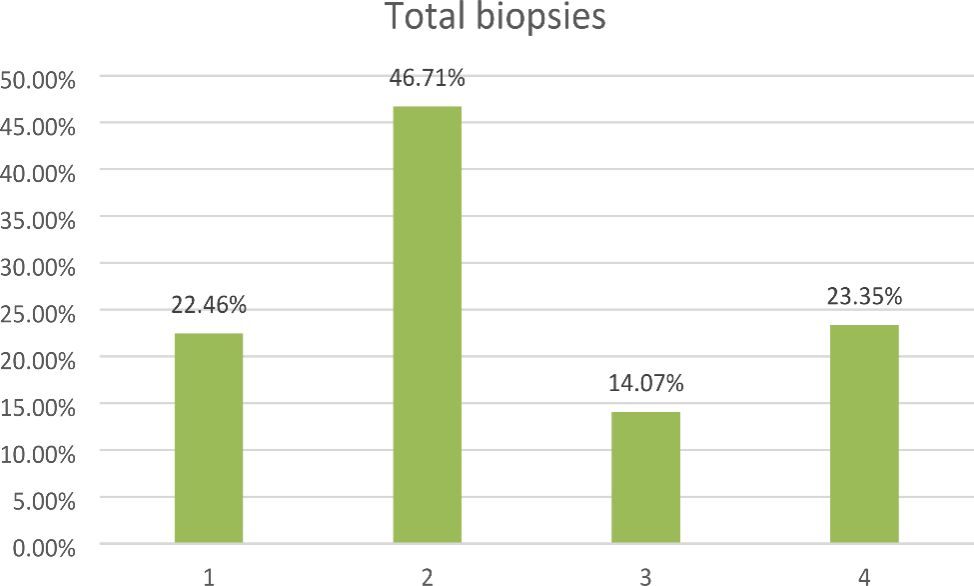
Overview total biopsies: 1 – *Staphylococcus aureus*, 2 – *Staphyloccocus epidermidis*, 3 – Enterococci, 4 – gram-negative bacteria

On average 3 ± 3.17 wound swabs and 4 ± 2.25 biopsies were performed in total per patient. During the radical sternectomy, 1 ± 0.77 wound swabs and 3 ± 1.54 biopsies were obtained on average. A pathogen could not be detected by wound swabs in 20 patients (22.99%) and by biopsy in 17 patients (19.54%). In total 340 wound swabs were taken from 87 patients. We identified *S. aureus* in 71 (20.88%), *S. epidermidis* in 35 (10.29%), *Enterococcus* spp. in 21 (6.18%) and gram-negative bacteria (GNB) in 125 (36.76%) wound swabs (Fig. 1). GNB comprised *Enterobacter cloacae, Escherichia coli, Klebsiella pneumoniae, Morganella morganii, Serratia marcescens, Pseudomonas aeruginosa, Proteus vulgaris* and *Proteus mirabilis*. In total 334 biopsies were taken for 87 patients. *S aureus* was found in 75 biopsies (22.46%), *S epidermidis* in 156 (46.71%), Enterococci in 47 (14.07%) and GNB in 78 (23.35%) (Fig. 2).

We also searched the medical charts before admission to our plastic surgery ward. In 69 patients (79.31%) a wound swab was made. In 53 cases (60.92%) a pathogen could not be identified, while *S. aureus* was isolated from 16 wounds (18.39%), *S. epidermidis* from six wounds (6.9%), *Enterococcus* spp. in three wounds (3.45%, GNB in seven wounds (8.05%) and two cases (2.3%) showed a polymicrobial infection (PMI) caused by *S. aureus* and GNB.

**Fig. 3:**
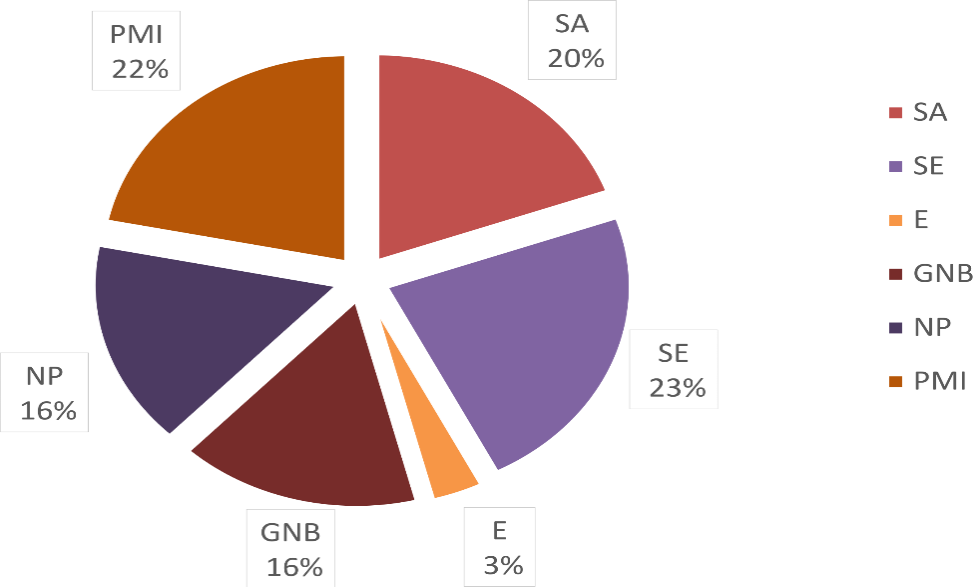
Pathogen diagnosis according to the clinical infectiology. SE – *Staphylococcus epidermidis*, SA – *Staphylococcus aureus*, GNB – gram-negative bacteria, E – Enterococci, PMI – polymicrobial infection, NP – no pathogen identified

**Fig. 4:**
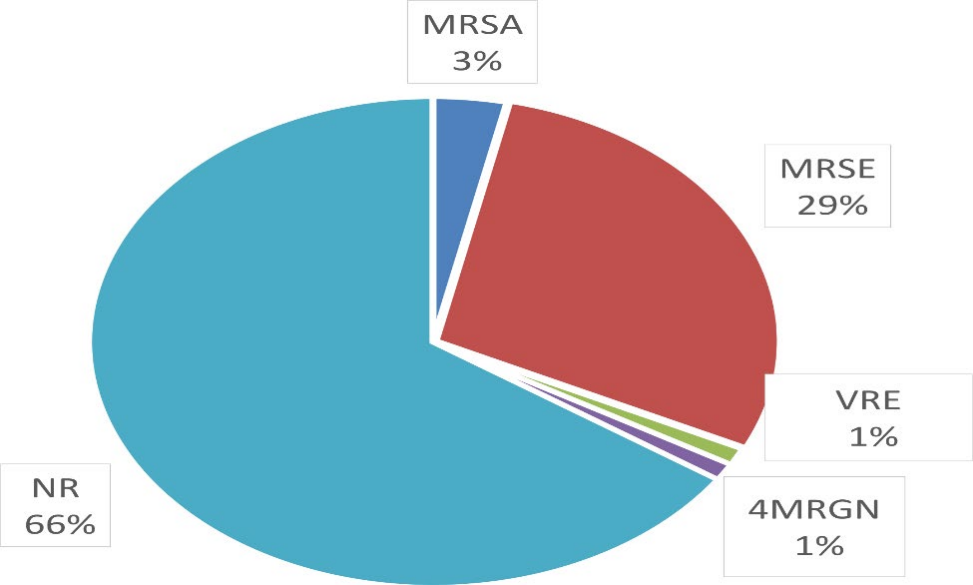
Incidence of multidrug-resistant pathogens. MRSA - Methicillin-resistant *Staphylococcus aureus*, MRSE: Methicillin-resistant *Staphylococcus epidermidis*, VRE: Vancomycin-resistant Enteroccoci, 4MRGN - multi-resistant gram-negative bacteria to 4 antibiotic groups

After gathering the microbiological data, there was a plethora of isolated bacteria from all the rest of the wound swabs and biopsies. Based on infectiological consultations, the most probable pathogens responsible for the DSWI were identified. The results showed that the DSWI in 20 patients (23%) was caused by *S. Epidermidis*, in 17 patients (19.54%) by *S aureus*, in 3 patients (3.45%) by *Enterococcus* spp., in 14 patients (16.09%) by .GNB, while in 14 patients (16,09%) no pathogen could be found. In 19 patients (21,84%) the sternal wound infection was caused by more than one pathogen defined as a PMI (Fig. 3). After evaluating the antibiograms, the resistant bacterial strains were identified. Methicillin-resistant *S. aureus* (MRSA) was found in only three cases (3,45%), Methicillin-resistant *S. epidermidis* (MRSE) in 25 cases (28,74%), Vancomycin-resistant Enterococci were found in one case (1,15%), while multi-resistant gram-negative bacteria to 4 antibiotic groups (4MRGN) were also found in one case (1,15%) (Fig. 4).

Fungi were sporadically isolated from the wounds. In sixteen wound swabs *Candida albicans*, in nine wound swabs *Candida glabrata* and in three wounds other types of candida species were found. The biopsies found four *C. albicans* and one *C. glabrata*. In two patients a mycotic infection was diagnosed and treated with Caspofungin. The first patient had a *C. albicans* infection superimposed on infection with GNB and SE. The wound swabs in this patient showed three times *C. albicans*, while the biopsies did not show any fungi growth. In the second patient, *C. glabrata* was found six times, *C. albicans* once and *Candida parapsilosis* once in the wound swabs, with negative biopsies, superimposed on a GNB infection.

The average hospital stay for monomicrobial infections was 29.93 ± 13.69 days and for polymicrobial infection was 37.47 ± 19.18 on average (p = 0.03). There was no statistically significant difference between the MMI and PMI groups considering the number of surgeries and the time elapsed until wound closure (p = 0.58 and 0.9) (Table 5).

**Table 5:** Statistical analysis of microbiological parameters

	Monomicrobial infection	Polymicrobial infection	p-value
Length of total antibiotic treatment	44.75 ± 25.87	61.29 ± 41.45	0.067
Hospital stay	29.93 ± 13.68	37.47 ± 19.18	**0.028**
Days until muscle flap coverage	9.24 ± 6.12	10.24 ± 7.19	0.58
Number of surgeries	2.07 ± 1.43	2.16 ± 1.5	0.89
Number of biopsies	4.42 ± 3.08	3.68 ± 1.97	0.54
Number of wound swabs	3.44 ± 1.98	5.58 ± 5.43	0.43
	Pathogen growth	No pathogen growth	
Number of biopsies	4.24 ± 2.22	2.18 ± 1.55	**< 0.001**
Number of wound swabs	4.22 ± 3.34	2.4 ± 1.45	**0.006**
	Staphyloccocus epidermidis	Other bacteria	
Number of biopsies	4.22 ± 1.99	3.62 ± 2.38	**0.05**
	Staphylococcus aureus	Other bacteria	
Number of wound swabs	4.76 ± 2.79	3.48 ± 3.28	**0.01**
	Enterococcus spp.	Other bacteria	
Number of wound swabs	6.4 ± 5.91	3.58 ± 2.51	**0.05**

Risk factors play a significant role in the evolution of DSWI. We found that ASA, smoking, alcohol, obesity, diabetes, endocarditis, atrial fibrillation, coronary heart disease, congestive heart failure, peripheral arterial occlusive disease, chronic obstructive pulmonary disease (COPD) and the type of heart surgery were not related to the development PMI or an infection relapse, while hyperlipidemia and myocardial infarction were associated with PMI in the univariate analysis (p = 0.04).

The median duration of intravenous antibiotic treatment was 24.62 (4–90) days, 23.54 (4–70) days of oral antibiotic treatment and 48.40 (10–159) days altogether. The length of antibiotic treatment for monomicrobial infections (MMI) was 22.68 ± 14.27 days intravenous and 44.75 ± 25.87 days in total and for PMI was 31.65 ± 22.29 days intravenous (p = 0.05) and 61.29 ± 41.45 in total (p = 0.07). The antibiotic treatment duration in patients with MRSA and MRSE as well as in patients who developed a relapse (chronic fistula) was not significantly longer. Empirical antibiotic therapy was initiated only when signs of systemic infection were detected and a de-escalation to targeted therapy occurred as soon as the antibiograms were available.

We could not find a correlation between the infection with multi-resistant bacteria or PMI and in-hospital death. Likewise, age, sex, BMI and comorbidities were not associated with in-hospital death.

There was no correlation between chronic infections and the infection with *S. aureus*, *S epidermidis*, Enterococcus spp., GNB or PMI. There was a significantly higher infection rate with *S. epidermidis* in men (44.78%) than in women (10%) (p = 0,007). Patients having DSWI with GNB had a significantly longer hospital stay (p = 0,031, Welch test).

The ASA score reflects the patient’s comorbidities and general status. All patients had ASA scores of 3 and 4. We could not find a significant correlation between the patient’s ASA score and one of the isolated pathogens.

We analyzed the relation between the wound swabs and biopsies and the isolation of pathogens from the wounds. We found a marginal significance when comparing wound swabs and biopsies in terms of unsuccessful isolation of pathogens (20 vs. 17, p = 0.05). The number of biopsies was positively correlated to the isolation of a pathogen (4.24 ± 2.22 vs. 2.18 ± 1.6, p < 0,001). The number of biopsies in patients diagnosed with *S. epidermidis* infection was higher than in patients where a *S. epidermidis* infection could not be determined (4.22 ± 2 vs. 3.62 ± 2.38, p = 0.052). The number of wound swabs appeared also to be correlated with the isolation of a pathogen (4.22 ± 3.34 vs. 2.40 ± 1.45, p = 0.011). In this case, the diagnosis of a *S. aureus* infection was related to the number of wound swabs (4.76 ± 2.79 vs. 3.49 ± 3.29, p = 0.011).

## Discussion

To isolate the etiological pathogen for DSWI, multiple wound swabs and bone biopsies need to be harvested. This leads in many cases to a plethora of isolated microorganisms, most of which have no clinical significance. The pathogens need to be differentiated from contamination from the wound margins. Skin flora including different strains of *S. aureus* and *S. epidermidis* may grow in different probes. Occasionally Enterococci and GNB also may signify only wound contamination, as these patients are usually hospitalized and the skin may be colonized with hospital germs. For the accurate identification of the causing pathogens, the whole microbiological probes and the antibiograms must be taken into consideration. The same strains of the pathogen and opportunistic bacteria should be encountered in several probes, especially from the biopsies, to be declared as the causal pathogen. Especially multiple strains of *S aureus* and *S epidermidis* with different antibiograms may be encountered in single probes, without clinical relevance. Our study showed that the increasing number of probes increases the chances of proper pathogen identification. Although tissue biopsies are expected to be more specific in pathogen identification, we could show that wound swabs still play a significant role. As wound swabs can be harvested in any situation from the open wound with little expenditure, we recommend that these should be performed whenever possible, while tissue biopsies are a prerequisite during surgical debridement.

After the first surgery with sternal removal, a high number of patients may develop pleural effusion. This may be attributed on one side to the systemic inflammatory process with capillary leakage and on the other side to the surgical trauma, where the sternum is removed and occasionally the pleural cavity may be opened. Although not as frequent, an inflammation of the lung parenchyma may also develop. This may or may be due to bacterial or fungal pneumonia. Systemic inflammation and impaired breathing in awake patients may facilitate this development. As these patients are multimorbid and will profit from a swift flap closure, it is in our practice not to postpone the flap surgery due to lung parenchymal changes or pleural effusion. The exception is the septic shock, when only a dressing change is performed to exclude a mediastinal focus of infection. After flap closure the lung function and the patients usually stabilize. The pleural effusions can be easily drained percutaneously or thorax drainage can be implemented during surgery when needed.

As described in the introduction, the comorbidities and several treatment factors have been proven to facilitate the development of DSWI [1, 4]. According to our study, these factors do not appear to influence the type of pathogen causing the infection. The existence of a PMI on the other hand extends the treatment time, without influencing the in-hospital death rate. The total number of surgeries and the time elapsed until wound closure were not different between MMI and PMI, as the only prerequisite for wound closure was a clean wound and not negative wound cultures. This confirms that our treatment strategy leads to swift infection control and a minimal in-hospital death rate. Hereby the radical debridement removes the dead tissue and the foreign bodies bearing biofilm while the comprehensive and accurate microbiological diagnosis facilitates the early targeted antibiotic therapy.

Although only 28,74% of the patients had an infection with GNB, the average hospital stay was longer in this cohort. Charbonneau et al. reported on a cohort of 309 patients with a similar incidence of DSWI with GNB. They found the female gender as a risk factor to develop a GNB infection and a higher 30-day mortality rate in these patients [10]. In our study, we found a trend toward more GNB infections in women (p = 0.063), whereby there were only 20 females in our cohort. There were no significant differences between the different pathogens and in-hospital mortality, whereas the global mortality rate was very low.

DSWI infections with fungi are rare. Ankan et al. reported in a review of 50 patients with *Candida* spp. mediastinitis. Only two patients had a superinfection with bacteria and *C. albicans* in this group, while most infections were caused by *C albicans* alone or as a superinfection, while *C. glabrata* was the pathogen in two cases. The treatment varied from medical treatment only to a combination of radical debridement, muscle flap closure and prolonged antimycotic therapy [11]. In our two cases the *C. albicans* and *C. glabrata* respectively could be successfully treated with our standard surgical treatment and targeted antimycotic therapy.

Compared to pathogenic *S. aureus*, *S. epidermidis* is an omnipresent human skin colonizer and has important roles in skin physiology [9]. Formerly described as an “accidental pathogen”, this perception has changed over time, *S. epidermidis* being the most frequent cause of implant-associated infections in the United States due to its remarkable biofilm-building ability [12]. The proven genetic flexibility of *S. epidermidis* allows intra- and inter-species genetic transfer, resulting in strains that are more pathogenic, build stronger biofilms and are increasingly multi-drug resistant [9]. While in the past *S. epidermidis* was not incriminated as a main pathogen in DSWI, some of the latest reports identify *S. epidermidis* as one of the main pathogens responsible for developing this condition, with a preponderance of 20 to 65% [2, 5, 13]. Our study found *S. epidermidis* to be the most frequent cause of DSWI, with a high percentage of methicillin-resistant strains. On the other hand, some current studies do not report *S. epidermidis* as a pathogen in DSWI [10]. While this could be explained by the local and regional spectrum of pathogens and their drug resistance, it could also be attributed to the diagnostic methods. In our study we could show that the diagnosis of *S. epidermidis* infection needs a higher number of bone tissue biopsies. At the same time the physician needs to consider *S. epidermidis* as a possible pathogen and not only as contamination from skin flora.

*S. aureus* is a notorious pathogen that has been long incriminated to play an important role in the development of DSWI. The nasal colonization with *S. aureus* is a recognized risk factor for developing SSI [1]. Especially the presence of MRSA in the nasal mucosa should be addressed by decontamination before performing cardiac surgery [14, 15]. *S. aureus* is known as the main pathogen in the development of osteomyelitis [16]. It has been proven to infect osteoblasts and osteoclasts and to alter bone metabolism. By replicating intracellularly, *S. aureus* can evade the immune system [7, 8, 17]. To address this issue, antibiotics with intracellular activity like oxacillin, moxifloxacin, linezolid, rifampicin or oritavancin are advocated [18], although the increasing number of MRSA strains complicate the treatment [16]. Our treatment strategy aimed at removing the whole infected sternal bone to facilitate the treatment and avoid relapses. The incidence of MRSA was relatively low, compared to the high number of MRSE. This can be attributed to the regional spectrum of bacterial drug resistance.

Although not the most frequent cause of DSWI, Enterococci represent a special challenge because of the frequent development of drug resistance [19]. The presence of VRE strains in the hospital should be considered when administering the perioperative antibiotic prophylaxis [2].

## Conclusion

Staphylococci remain the main pathogen in DSWI, followed by gram negative bacteria and *Enterococcus* spp. MRSE were the most frequently encountered resistant strains. Polymicrobial infections represent about a fourth of DSWI. Although the number of wound swabs and tissue biopsies correlates with accurate pathogen isolation, in 16% of the cases a pathogen could not be identified, which indicates the need for further improving the microbiological diagnostic techniques. The resistance spectrum of the pathogens and the antibiotic treatment duration had no significant impact on the infection relapse or mortality. With radical surgical treatment, the role of prolonged antibiotic treatment remains unclear and should be evaluated in future prospective randomized studies.

## Data Availability

The datasets used and/or analyzed during the current study are available from the corresponding author upon reasonable request.

## Declarations

### Author‘s information

OB is a plastic surgeon, currently assistant medical director at the Center of Orthopedics, Trauma and Plastic Surgery, specializing in reconstructive surgery, with a broad experience in the treatment of deep sternal wound infections. He is a member of the German societies of plastic surgery, hand surgery and surgery.
